# Nitroreduction of flutamide by *Cunninghamella elegans* NADPH: Cytochrome P450 reductase

**DOI:** 10.1016/j.bbrep.2022.101209

**Published:** 2022-01-17

**Authors:** Mohd Faheem Khan, Cormac D. Murphy

**Affiliations:** UCD School of Biomolecular and Biomedical Science, University College Dublin, Belfield, Dublin 4, Ireland

**Keywords:** Microbial model, Drug metabolism, CYP reductase, Biotransformation

## Abstract

The microbial model of mammalian drug metabolism, *Cunninghamella elegans*, has three cytochrome P450 reductase genes in its genome: g1631 (CPR_A), g4301 (CPR_B), and g7609 (CPR_C). The nitroreductase activity of the encoded enzymes was investigated via expression of the genes in the yeast *Pichia pastoris* X33. Whole cell assays with the recombinant yeast demonstrated that the reductases converted the anticancer drug flutamide to the nitroreduced metabolite that was also produced from the same substrate when incubated with human NADPH: cytochrome P450 reductase. The nitroreductase activity extended to other substrates such as the related drug nilutamide and the environmental contaminants 1-nitronaphthalene and 1,3-dinitronaphthalene. Comparative experiments with cell lysates of recombinant yeast were conducted under aerobic and reduced oxygen conditions and demonstrated that the reductases are oxygen sensitive.

## Highlights

• Three cytochrome P450 reductase genes from *Cunninghamella elegans* were heterologously expressed in *Pichia pastoris.*
• TThe enzymes displayed nitroreductase activity towards flutamide, which is analogous to human cytochrome P450 reductase.
• The enzymes are oxygen sensitive, which is also a property shared with the human enzyme.
• Other nitro-containing substrates can be reduced by the fungal enzymes.

## 1 Introduction

Microbial drug biotransformations can be employed in the drug development process for the production of hard-to-synthesise metabolites [1] and to improve drug design [2]. Some species of the filamentous fungus *Cunninghamella*, in particular *C. elegans and C. echinulata*, are noted for their ability to mimic the mammalian metabolism of drugs and other xenobiotics [3,4]. These organisms can catalyse phase I (oxidative) and phase II (conjugative) reactions and the enzymes, such as cytochromes P450 (CYPs), glutathione *S*-transferase and glucosyl transferase, have been experimentally assayed [5]. Recently, Palmer-Brown et al. [6] identified the CYPome of *C. elegans,* which comprises 32 putative CYPs, two of which (5313D1 and E1) have a high homology to the mammalian CYP3A4, which is centrally involved in drug detoxification in mammals. CYP5313D1 was heterologously expressed in *Pichia pastoris* and the recombinant yeast could hydroxylate the drug flurbiprofen generating the phase I mammalian metabolite 4′-hydroxyflurbiprofen, confirming the CYP as a key enzyme in *C. elegans* for xenobiotic biotransformation. Other xenobiotic-transforming CYPs are likely to be present in *C. elegans* and the study by Palmer-Brown et al. has provided access to the different genes that are responsible these reactions, enabling deeper analysis of their characteristics and broadening potential application to drug metabolite production.

**Fig. 1:**
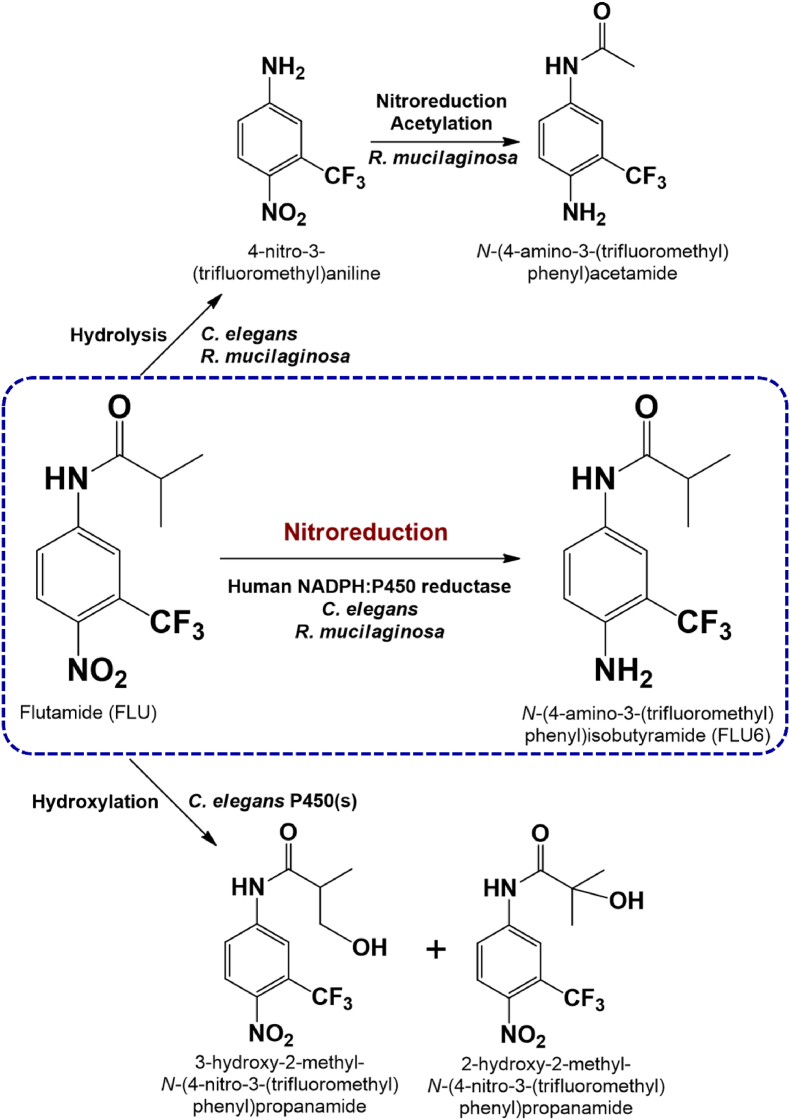
Key biotransformations of FLU in humans and fungi.

Flutamide (FLU) is an antiandrogenic drug used to treat prostate cancer and is metabolised in humans via hydroxylation, hydrolysis, *N*-acetylation and nitroreduction [7,8]. Amadio et al. [9] demonstrated that the drug can be biotransformed by *C. elegans* to yield the same metabolites as those found in humans (Fig. 1). One other fungus, *Rhodotorula mucilaginosa*, is also known to catabolise FLU [10], but does not produce the hydroxylated metabolites and elaborates one additional *N*-acetylated product, *N*-[4-amino-3-(trifluoromethyl)phenyl]acetamide. Whilst the main phase I metabolism of FLU in humans is hydroxylation catalysed by CYP1A2 [8], Wen et al. [11] studied the nitroreduction of the drug, since this biotransformation is potentially the cause of hepatotoxicity. These researchers demonstrated that the human NADPH: cytochrome P450 reductase (CPR), rather than CYP activity, directly reduced the nitro group of the drug, yielding *N*-[4-amino-3-(trifluoromethyl)phenyl]isobutyramide (FLU-6, Fig. 1).

A CPR gene from *C. elegans* was identified by Yadav and Loper [12] after screening a phagemid genomic library of the fungus using a 288 bp amplicon previously obtained using degenerate primers based on conserved sequences of other CPRs. The complete gene sequence was determined from a single clone carrying the entire CPR gene. Detailed sequence characterisation of the gene enabled a protein sequence to be deduced, which revealed an N-terminal transmembrane region and the functional domains involved in cofactor and CYP binding. Phylogenetic comparisons revealed that the protein has 41–42% homology to animal CPRs. Transcriptional analysis via Northern blot showed that expression of the gene in the fungus is induced by n-tetradecane; later studies found it is also induced by other xenobiotics such as carvediol [13], chloroxylenol [14], steroids and polyaromatic hydrocarbons [15].

Analysis of the CYPome in *C. elegans* by Palmer-Brown et al. [6] also identified the CPR gene (g4301) originally sequenced by Yadav and Loper [12], and uncovered the presence of two additional putative CPRs, g7609 and g1631. No *in vitro* assessment of the *C. elegans* CPR activity has yet been undertaken. In this paper we describe the cloning of the three CPR genes from *C. elegans* in the yeast *Pichia pastoris*, and demonstrate that all of them can reduce the nitro-group of FLU in the same manner as human CPR. Furthermore, the original protein identified by Yadav and Loper is oxygen sensitive, which is also a characteristic of human CPR, and can reduce other nitro containing drugs and xenobiotics.

## 2 Materials and methods

### 2.1 Materials

Culture media, biochemical substrates and derivatising reagents were from Sigma-Aldrich (Arklow, Ireland). Invitrogen™ EasySelect™ Pichia Expression Kit, Zeocin™, EZ-Run™ Prestained *Rec* Protein Ladder and HisPur™ Ni-NTA resin (Thermo Fisher Scientific) were procured from Fisher Scientific (Dublin, Ireland). QIAprep Spin Miniprep Kit was purchased Qiagen (USA). Restriction enzymes (New England Biolabs) were obtained from Brennan and Company (Dublin, Ireland). Amicon Ultra-15 10K Centrifugal Filter Units (Merck Millipore) were acquired from VWR International (UK). Other solvents and chemicals used in the present study were of molecular biology and analytical grade.

### *2.2* *Growth of* Cunninghamella elegans *and biotransformation of flutamide*

The method for culturing *Cunninghamella elegans* DSM1908 was the same as Khan and Murphy [16]. Briefly, the fungal inoculum was prepared by cultivating the fungi on sabouraud dextrose agar 120 h at 28 ᵒC followed by homogenisation of grown mycelium on the agar with 100 mL autoclaved water using hand blender. For suspended cultures, 5 mL of inoculum was added to 45 mL SDB were incubated at 28 °C with 150 rpm shaking for 72 h.

Flutamide was dissolved in DMF and introduced (0.1 mg/mL) to the 72 h-grown cultures of *C. elegans*, which was further incubated for 72 h at 28 ᵒC with 150 rpm agitation. The fungal biomass was separated by centrifugation at 9000 rpm for 10 min and the supernatant was used for metabolite extraction with ethyl acetate. A fungal control experiment was performed without flutamide.

### 2.3 Gas chromatography-mass spectrometry (GC-MS) analysis

The method employed for the GC-MS analysis was that used by Khan and Murphy [17]. Briefly, the samples were dried under N_2_ gas and silylated using 50 μL MSTFA at 100 °C for 45 min, then ethyl acetate was added to adjust the final volume to 0.5 mL. Samples were analysed in the split mode (20:1) using a 7890B N Agilent GC system equipped with a 30 m × 0.25 mm × 0.33 μm HP-5MS capillary column and a 5977A mass-selective detector; the oven temperature was 90 °C for 3 min then raised to 300 °C at 10 °C/min.

### 2.4 Cloning, expression and purification of CPRs

The codon optimised (OptimumGene™) genes for g1631 (CPR_A), g4301 (CPR_B), and g7609 (CPR_C) were synthesised by Genscript Biotech (Netherlands) and cloned in pPICZ A vector for expression in *Pichia pastoris* X-33. Sequences of the proteins and genes are given in Table S1. Clones were confirmed by restriction digestion using KpnI and XhoI (Fig. S1A) and sequence analysis. The cloned vectors were transformed into electro-competent *P. pastoris* X-33 cells using 0.2 cm electroporation cuvettes at 1500 V according to EasySelect™ Pichia Expression Kit manual. The transformed colonies were screened on YPD(S) agar plates with increased concentrations of Zeocin (100–2000 μg/mL) by growing for 3–4 days at 30 °C.

For expression of CPRs, a single colony of recombinant yeast was grown in baffled 250 mL Erlenmeyer flasks containing 40 mL sterile BMGY (20 g/L peptone, 10 g/L yeast extract, 13.4 g/L yeast nitrogen base without amino acids, 4 mg/L biotin, 0.1 M potassium phosphate pH 6.0, 1% glycerol) that were incubated for 24 h at 30 °C with shaking at 280 rpm. The cells were harvested by centrifugation (4000 rpm, 10 min and 4 °C); the supernatant was decanted and the pellet resuspended in 40 mL fresh sterile BMMY containing 1% methanol instead of glycerol. The cultures were incubated for 72 h at 30 °C and 280 rpm agitation and additional methanol was added at 24 h intervals.

For purification of CPRs, the methanol-induced recombinant yeast cells were harvested and disrupted by incubating in lysis buffer (0.1 M NaOH, 0.05 M EDTA, 2% SDS and 2% DTT) at 90 °C for 10 min, whereupon 0.2 M acetic acid was added and incubated for further 10 min. The lysates were sonicated (Fisherbrand model 120 sonic dismembrator) at 35% amplitude, 5 s pulse on and 10 s pulse off for total 5 min on ice. The lysate was centrifuged at 16000 rpm, 4 °C for 10 min and the supernatant loaded on a 1 mL Ni-NTA column. After washing with 50 mM sodium phosphate buffer (pH 8.0) containing 20 mM imidazole the CPR was eluted using same buffer containing 250 mM imidazole. The purified CPRs were concentrated using Amicon Centrifugal Filters and purity confirmed by 10% SDS-PAGE (Fig. S1B).

### 2.5 Determination of nitroreductase activity

The nitroreductase activity was determined by whole cell and cell free assays. Recombinant *P. pastoris* were cultured in BMMY as described above and 0.1 mg/mL of substrate (flutamide, nilutamide, 1-nitronaphthalene and 1,3-dinitronaphthalene) was added 24 h after induction with methanol and incubated for another 48 h at 30 °C and 280 rpm agitation. The cultures were extracted with ethyl acetate and the derivatised extracts were analysed by GC-MS. To determine the effect of a selective CPR inhibitor on biotransformation, α-lipoic acid (0.5–10 mM) was incubated with whole cells for an hour prior to adding substrate. Percentage inhibition was determined by comparing peak areas of the product in the presence of α-lipoic acid to those of control experiments to which no inhibitor was added.

Activity was measured in cell free extracts by harvesting methanol-induced cells, resuspending them in 50 mM sodium phosphate buffer (pH 8.0) and sonicating as described above. The sonicate was centrifuged 16000 rpm, 4 °C for 10 min, and the supernatant purged with N_2_ gas for 30 min to create a reduced oxygen environment. Substrate (0.1 mg/mL) and NADPH (10 mM) was added to the cell free extract and incubated at 30 °C for 6 h. The assay mixtures were extracted with ethyl acetate and the derivatised extracts analysed by GC-MS.

## 3 Results

### 3.1 C. elegans CYP reductases have nitroreductase activity

**Fig. 2:**
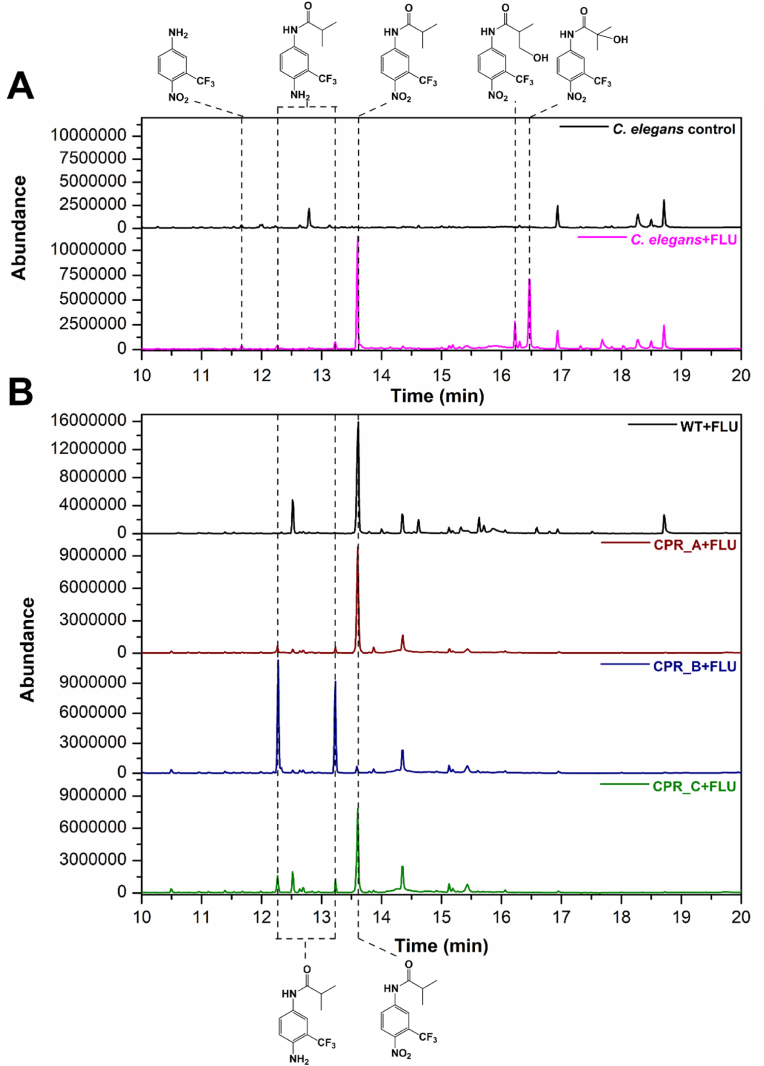
Biotransformation of FLU by *C. elegans* (A) and the recombinant *P. pastoris* expressing the different CPRs (B). The structures of the metabolites are shown; the nitroreduced metabolite (FLU6) was derivatised with one or two trimethylsilyl groups, hence two peaks are apparent.

Our previous study investigating the metabolism of FLU by *C. elegans* [9] revealed that one of the minor products was FLU6 (Figs. 1 and 2A). This product was also identified by Wen et al. [11] upon incubating purified recombinant human CPR with FLU, thus we speculated that one or more of the CPRs in *C. elegans* might have the same nitroreductase activity. To investigate this further, we cloned each of the CPR genes in *P. pastoris* using the pPICZ A plasmid without the N-terminal signal peptide so that the protein would remain in the cell, since the CPRs are predicted to have an N-terminal transmembrane segment. After induction of protein expression by methanol, FLU was added to the cultures and after 48 h incubation the cultures were extracted with ethyl acetate. After drying the solvent, the residue was silylated and analysed by GC-MS. Wild type *P. pastoris* did not transform FLU (Fig. 2B) as the drug was readily detected eluting after 13.6 min and no new peaks were observed in the chromatogram compared with yeast that was incubated without FLU. In contrast, the recombinant yeast expressing the different CPRs (g1631, g4301 and g7609; for ease of identification now labelled CPR_A, _B and _C, respectively) transformed FLU to FLU-6, which appeared as two peaks in the chromatogram, eluting after 12.3 and 13.2 min (Fig. 2B). The molecular ions (M^+^) of the products were *m/z* 318 and 390, respectively, since the metabolite was derivatised with one or two trimethylsilyl groups. Thus, all three CPRs in *C. elegans* have nitroreductase activity similar to that of the human enzyme, but CYP_B appeared to be most active based on product peak areas. This enzyme was evaluated for inhibition by α-lipoic acid (0.5–10 mM), which is a reversible inhibitor of human CPR [18], and dose-dependent inhibition was observed with flutamide and the related drug nilutamide (see below) as substrates (Table 1 and Fig. S2).

**Table 1:** Inhibition of flutamide and nilutamide biotransformation in recombinant *P. pastoris* expressing CYP_B using α-lipoic acid. Control experiments were conducted in the absence of the inhibitor.

α-lipoic acid concentration (mM)	Inhibition (%)	
	Flutamide	Nilutamide
0.5	19.4 ± 2.7	23.9 ± 4.8
1	32.5 ± 3.3	41.0 ± 2.0
2	49.9 ± 1.9	56.7 ± 3.1
5	99.0 ± 2.7	84.4 ± 7.2
10	99.7 ± 4.1	98.6 ± 1.7

### 3.2 C. elegans CPR can reduce other nitro-containing xenobiotics

**Fig. 3:**
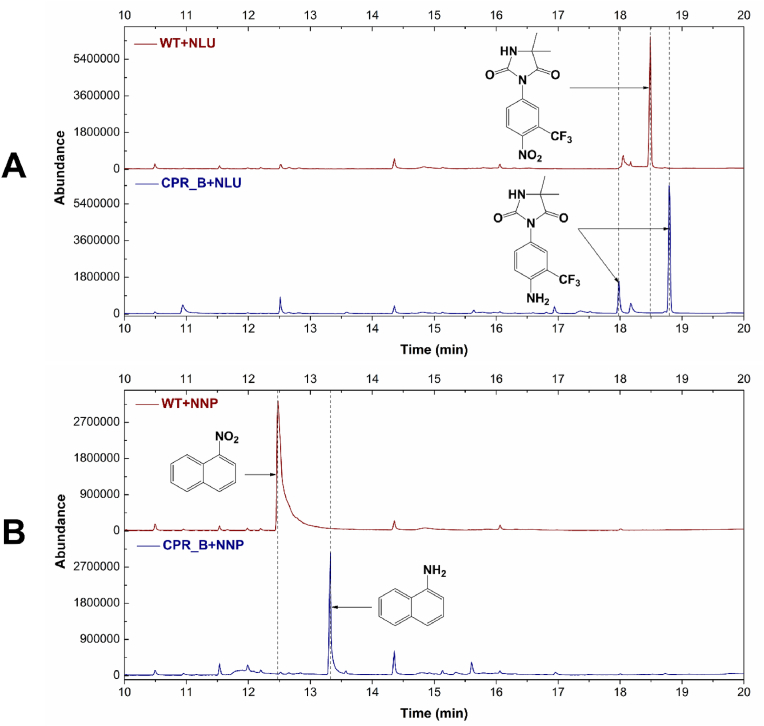
Biotransformation of the nitroaromatic xenobiotics nilutamide (NLU) and 1-nitronaphthalene (NNP) by *C. elegans* CPR_B expressed in *P. pastoris*. The mass spectra of the products are shown in Figs. S3 and S4.

To determine if the reductases can reduce nitro groups on other substrates, recombinant *P. pastoris* expressing CPR_B from *C. elegans* was incubated with nilutamide, which is a drug related to flutamide. Analysis of extracts by GC-MS revealed no biotransformation in the wild-type yeast, but in the recombinant yeast a large product peak was observed (retention time 18.8 min), with the expected mass (M^+^ *m/z* = 359) of the silylated amino derivative (Fig. 3A). A smaller product peak eluting at 18.0 min had the expected mass of the doubly silylated metabolite (M^+^ *m/z* = 431). Furthermore, 1-nitronaphthalene, which is an environmental contaminant arising from diesel fumes, was not transformed by the wild type, but was reduced by the recombinant yeast to 1-aminonaphthalene (Fig. 3B). The related compound 1, 3-dinitronaphthalene was also transformed by the recombinant yeast, yielding a product in which one of the nitro groups was reduced to amino (Fig. S5). Based on the mass spectrum only, it was not possible to tell which of the nitro groups was reduced, but the presence of only one product suggests that only one of the groups was transformed.

### 3.3 C. elegans CPR is oxygen sensitive

**Fig. 4:**
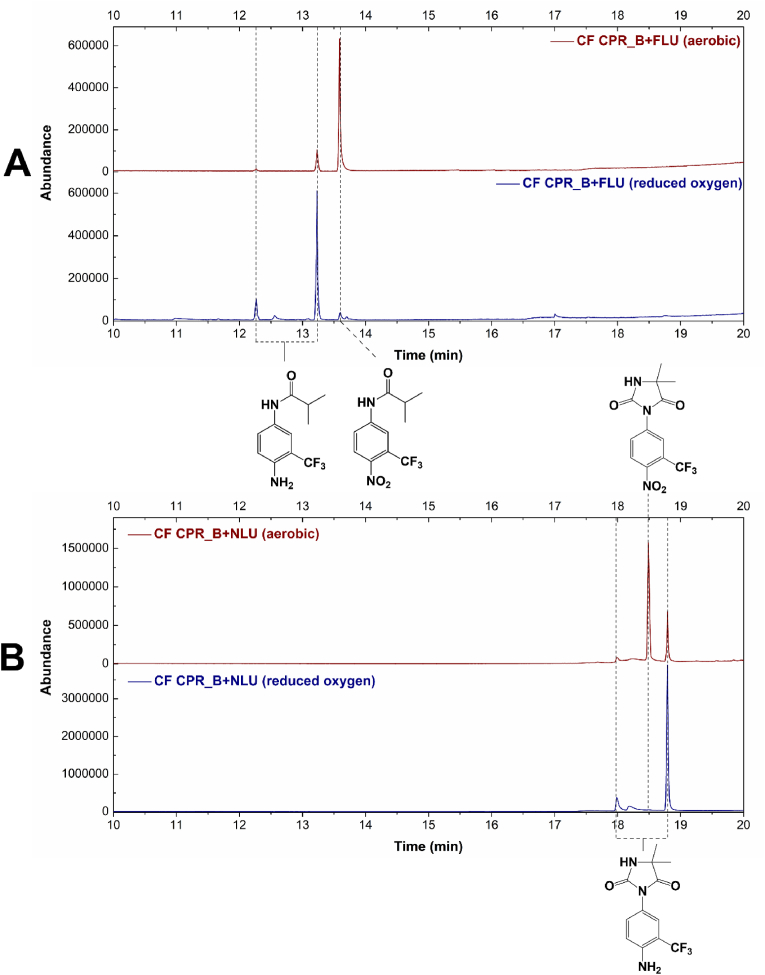
TIC of extracts from cell free extracts of *P. pastoris* expressing CPR_B incubated with FLU (A) and NLU (B) under aerobic (red line) and reduced oxygen (blue line) conditions.

Wen et al. [11] demonstrated that human CPR-catalysed nitroreduction of FLU was oxygen sensitive, since the reaction was approx. 129 times faster when the assay mixtures were purged with argon. To investigate if the *C. elegans* CPR was similarly oxygen sensitive, crude cell free extracts of recombinant *P. pastoris* expressing the different CPRs were prepared by sonication, purged with nitrogen and incubated with FLU. After 6 h the assays were extracted with ethyl acetate and the organic fraction was analysed by GC-MS. FLU-6 was readily detected in lysates containing CPR_B, whereas the extract from a control experiment conducted with non-purged lysate revealed no FLU-6 and only the starting substrate (Fig. 4A). The lysates containing CPR_A and CPR_C did not show any biotransformation under either condition (not shown). The experiment was also repeated with nilutamide as the substrate (Fig. 4B) and showed similar results, with complete transformation of the substrate under reduced oxygen conditions and only partial transformation under aerobic conditions with CPR_B. Using this substrate, the other *C. elegans* CPRs produced a small amount of product under reduced oxygen conditions (Fig. S6), but no product was detected in cell free extracts that were not purged with nitrogen.

## 4 Discussion

Many parallels have been demonstrated between the metabolism of xenobiotics in mammals and fungi, in particular species of the genus *Cunninghamella*. These similarities have been shown most convincingly with phase I (oxidative) reactions with drugs, which are catalysed by CYPs [19]. Since the initial discovery of mammalian-equivalent metabolites of xenobiotics in fungi [20], dozens of studies have been conducted using whole cells. *In vitro* assessment of CYP activity in *C. elegans* has been more challenging, although some progress has been made more recently with the heterologous expression of CYP5313D1 and measurement of monooxygenase activity [6]. A feature of fungal CYPs is the requirement for CPRs to transfer electrons from NADPH to the haem. The interaction between CPRs and CYPs is important not only for overall activity, but also for the types of reactions that are catalysed [21,22]. Yadav and Loper [12] previously identified the gene coding for a CPR (named CPR_B in the present study) and subsequently Palmer-Brown et al. [6] reported the presence of two others (CPR_A and C). In the former study it was reported that the protein had the highest homology to the CPRs in *Aspergillus niger* (46%) and animals (41–42%). An updated *in silico* analysis has identified that CPRs in filamentous fungi such as *Absidia repens* and *Lichtheimia corymbifera* have over 70% identity with CPR_B in *C. elegans*. CPR_A and C have 66% and 47% identity, respectively, to CPR_B, and have a roughly similar homology with human CPR.

While the role of CPRs as redox partners of fungal CYPs has been demonstrated [[22], [23], [24]], their stand-alone activities in relation to drug biotransformation have not been explored. Human CPR has nitroreductase activity towards the antiandrogen drug flutamide, which yields a metabolite (FLU6) potentially responsible for hepatotoxicity. Since *C. elegans* is a model of mammalian drug metabolism, and whole cells of the fungus also produce FLU6 as one of the metabolites of FLU [9], it is possible that the fungal CPRs might also have nitroreductase activity. We have now shown that the three putative CPRs present in *C. elegans* can also reduce flutamide, and other nitro aromatic substrates, when the genes are heterologously expressed in *P. pastoris*. Furthermore, other characteristics of the human CPR are shared by the *C. elegans* CPRs: the fungal enzymes are similarly inhibited by α-lipoic acid and after purging *P. pastoris* cell extracts with nitrogen prior to addition of substrate, it was shown that CPR is sensitive to oxygen. Thus, the capacity of the fungus to act as a model of mammalian drug metabolism extends beyond CYPs to the CPRs. Given the closer phylogenetic relationship of other fungal CPRs to CPR_B in *C. elegans*, compared to the human protein, it is possible that nitroreductase activity is a characteristic that is common in these organisms.

## Declaration of competing interest

The authors declare that they have no known competing financial interests or personal relationships that could have appeared to influence the work reported in this paper.

## Data Availability

Data will be made available on request.
