# Supplementary material for: Nitroreduction of flutamide by Cunninghamella elegans NADPH: Cytochrome P450 reductase
Source: Biochem Biophys Rep. 2022 Jan 17;29:101209. doi: 10.1016/j.bbrep.2022.101209 (PMC8783101; doi:10.1016/j.bbrep.2022.101209)
Supplement: Multimedia component 1 [file mmc1.docx]

**Nitroreduction of Flutamide by *Cunninghamella elegans* NADPH: Cytochrome P450 Reductase**

Mohd Faheem Khan and Cormac D. Murphy*

UCD School of Biomolecular and Biomedical Science, University College Dublin, Belfield, Dublin 4, Ireland

**Supplemental information**

Figure S1. Confirmation by double digestion of successful cloning of CPR genes (A) and their expression (B) in *P. pastoris.*


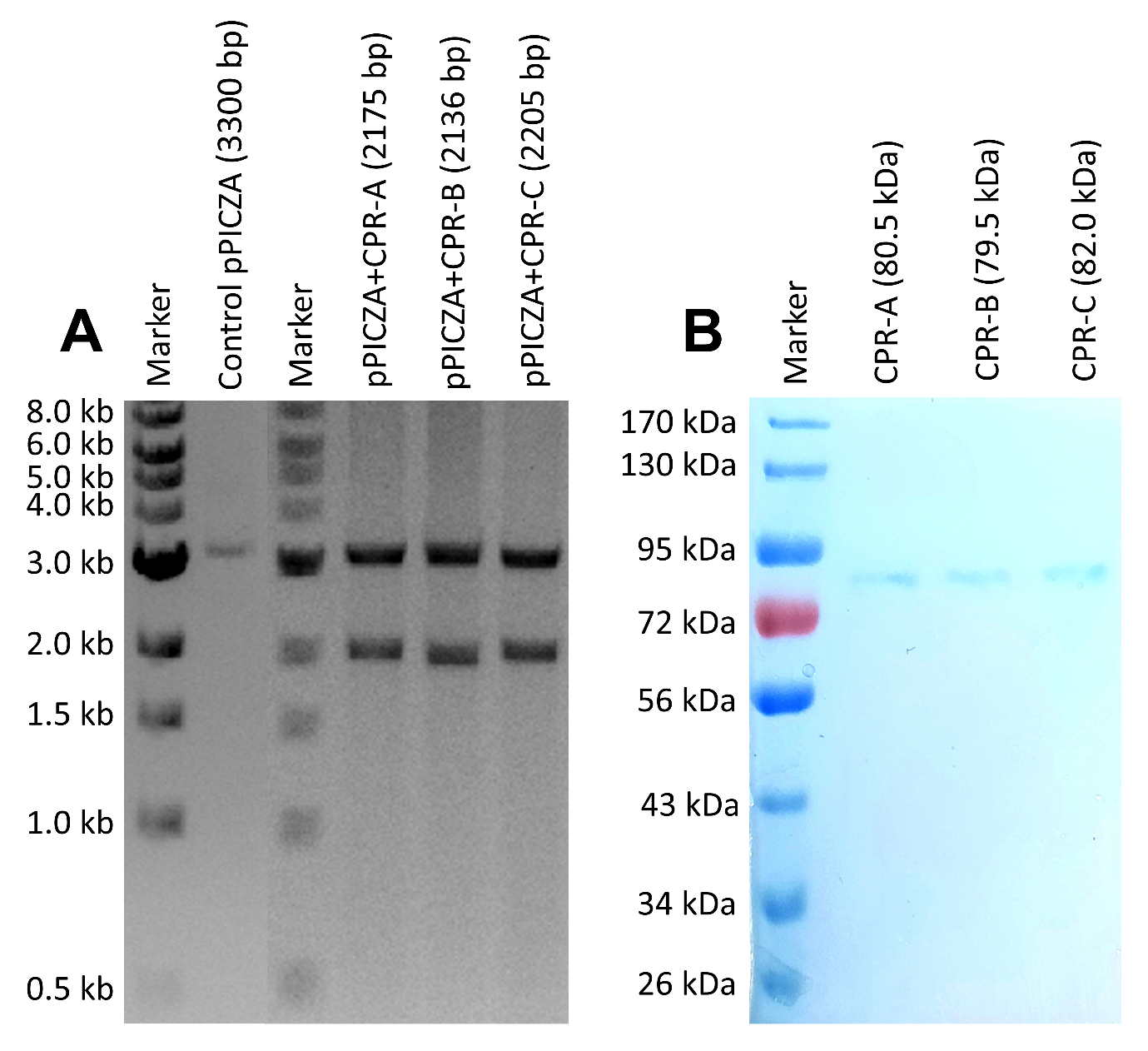


Figure S2. Inhibition of flutamide (A) and nilutamide (B) nitroreduction by α-lipoic acid. The dashed lines show the elution of the product peaks.


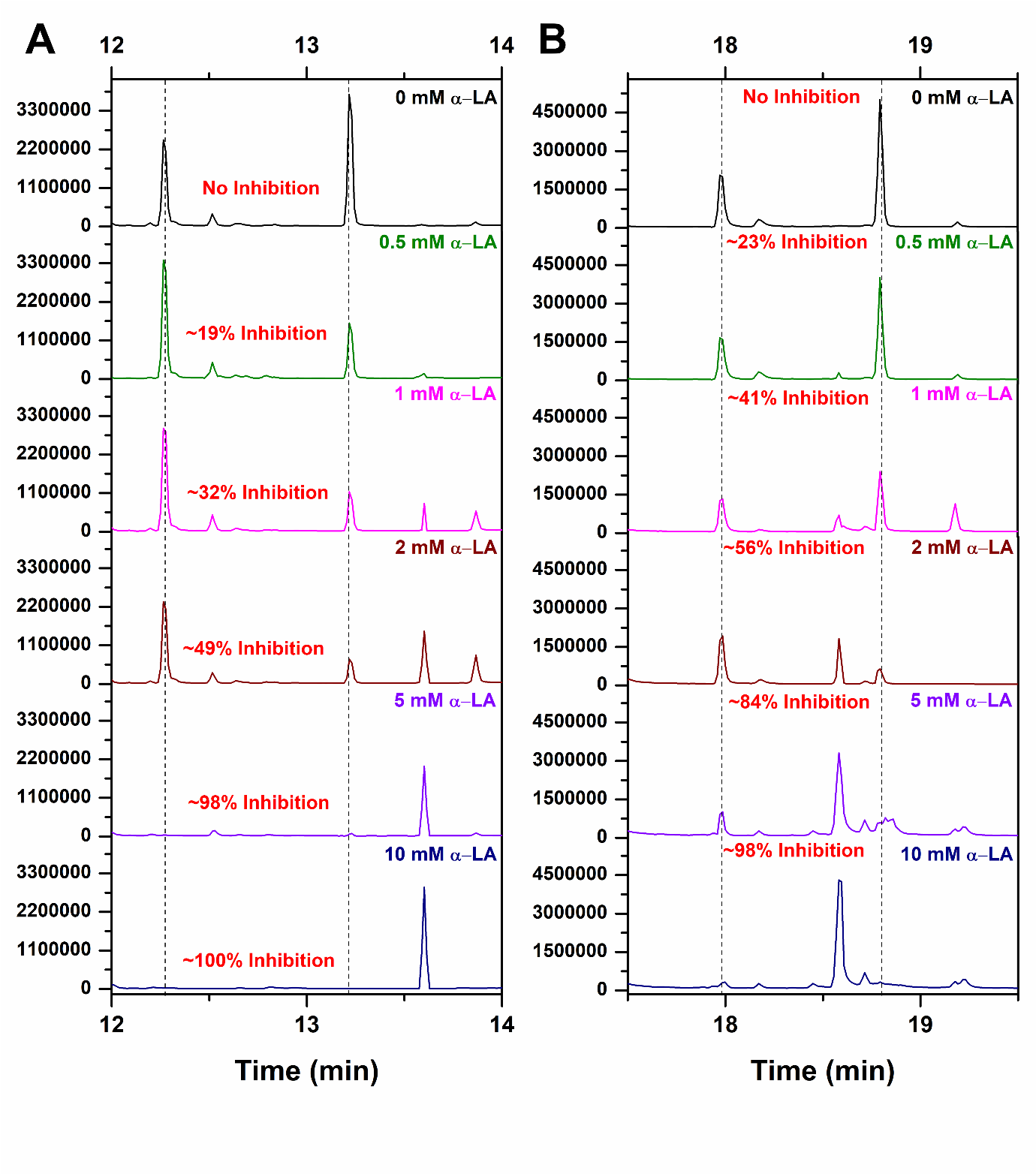


Figure S3. Mass spectra of silylated nilutamide (A) and singly (B) and doubly (C) silylated nitroreduced metabolites produced upon incubation of the drug with recombinant *P. pastoris* (TMS=trimethylsilyl).


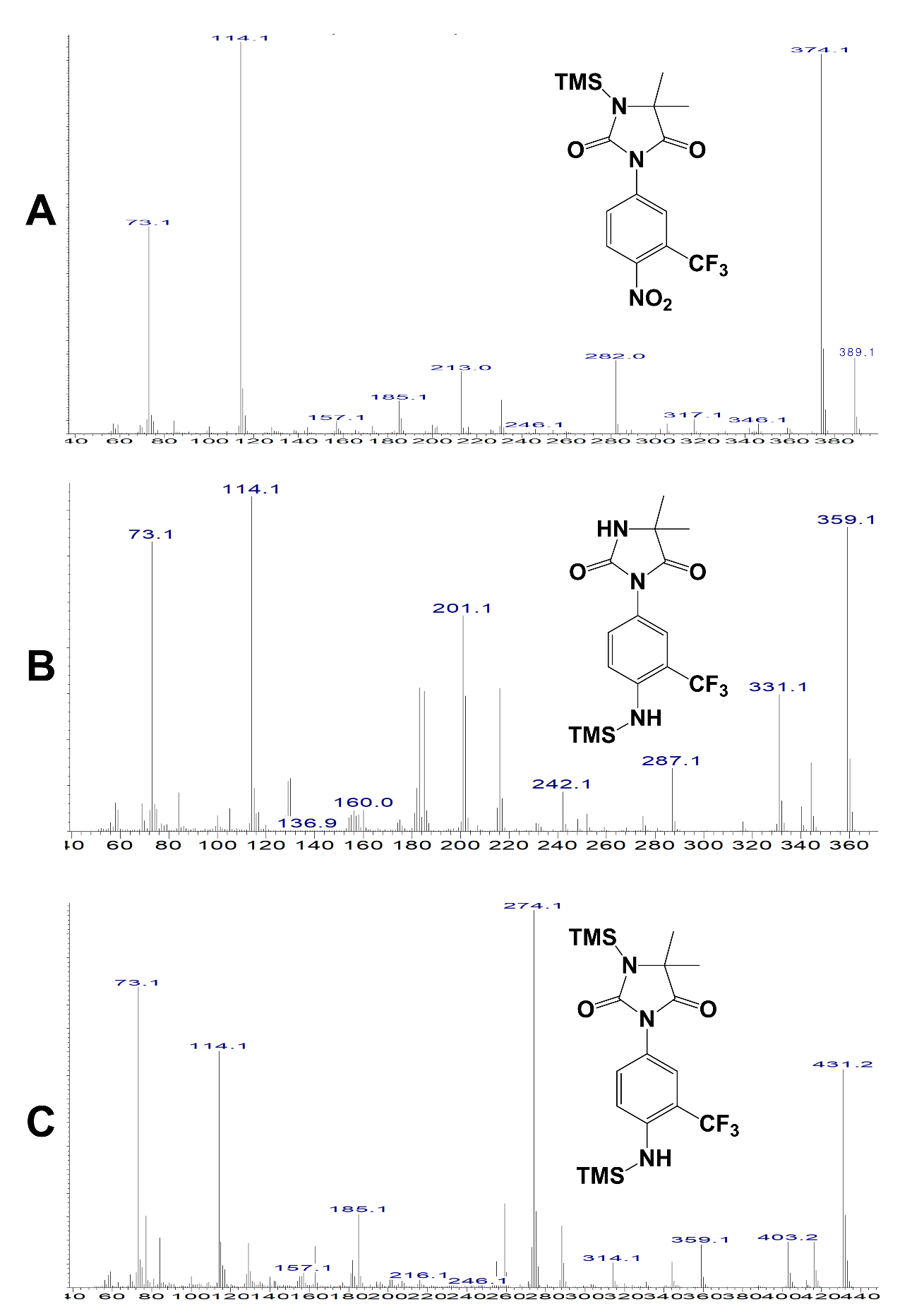


Figure S4. Mass spectra of nitronaphthalene (A) and 1-aminonaphthalene (B).


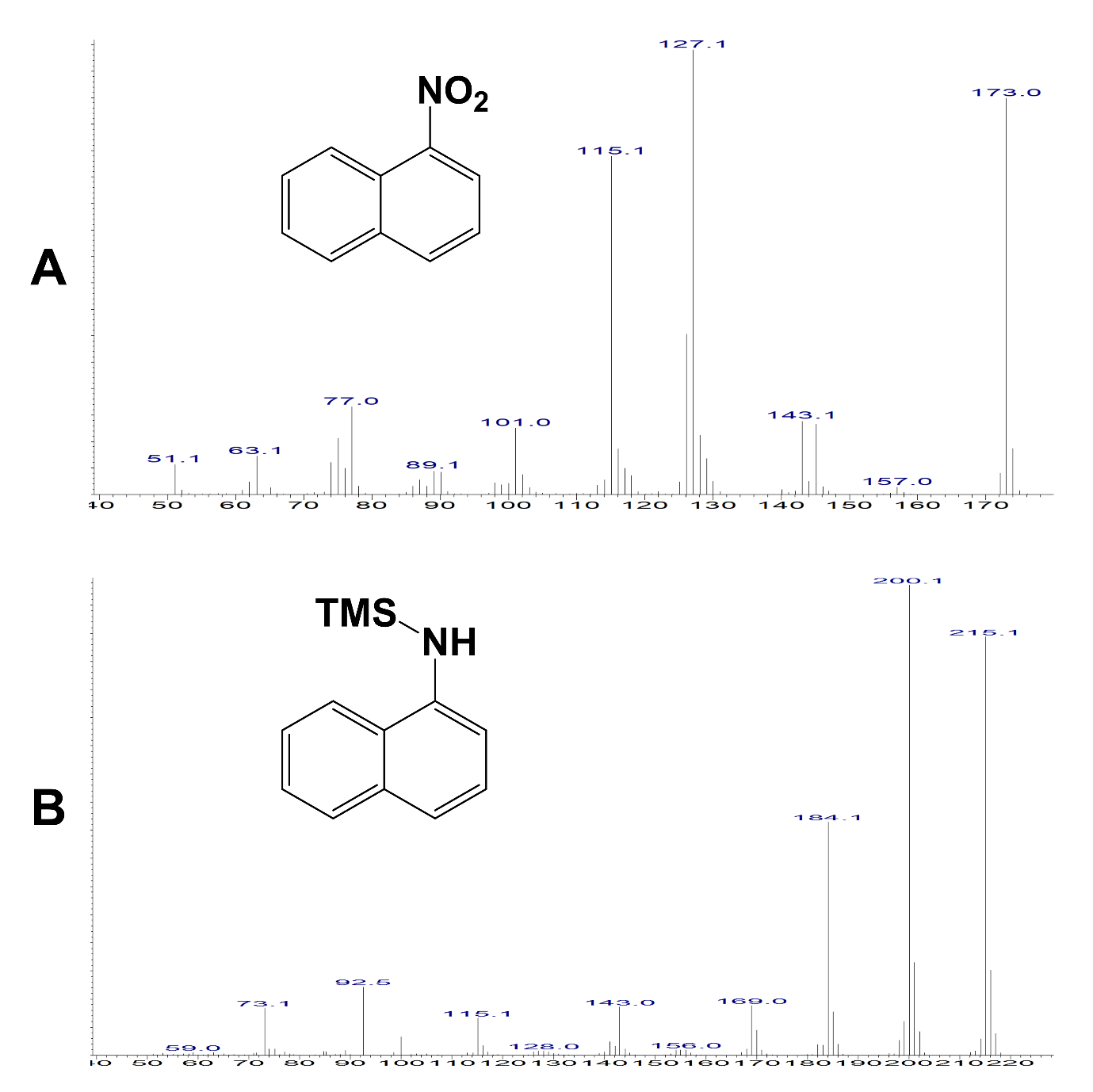


Figure S5. Total ion chromatogram (A) of the biotransformation of 1,3-dinitornaphthalene by recombinant *P. pastoris*. Mass spectra of 1,3-dinotronaphthalene (B) and its reduced product derivatised with one (C) are also shown. It was not possible to determine which of the nitro groups was reduced.


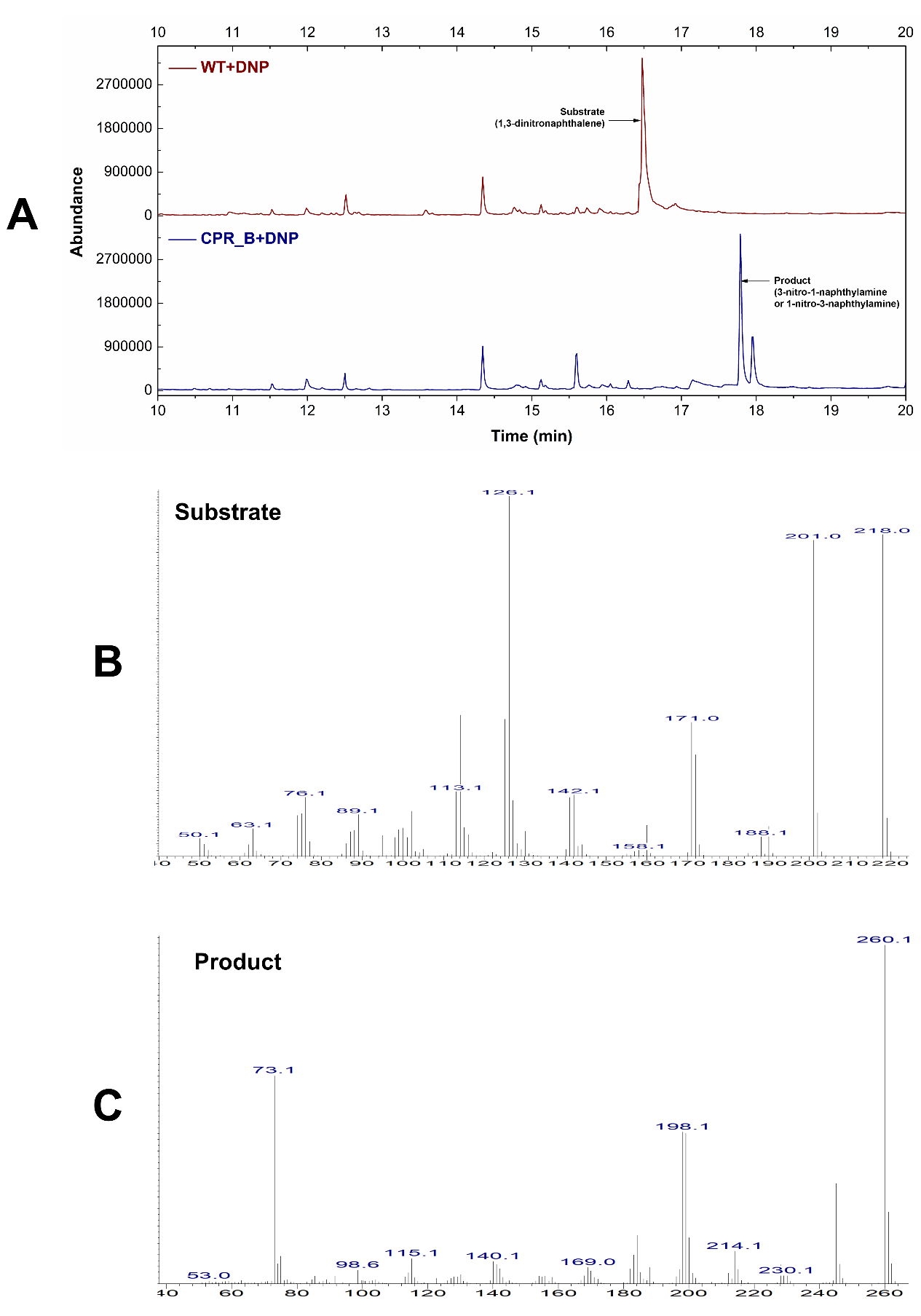


Figure S6. The effect of oxygen on in vitro biotransformation of nilutamide (NLU) by CPR_A (A) and _C (B). The chromatograms show the formation of a small amount of product eluting after 18.8 min only under reduced oxygen conditions.


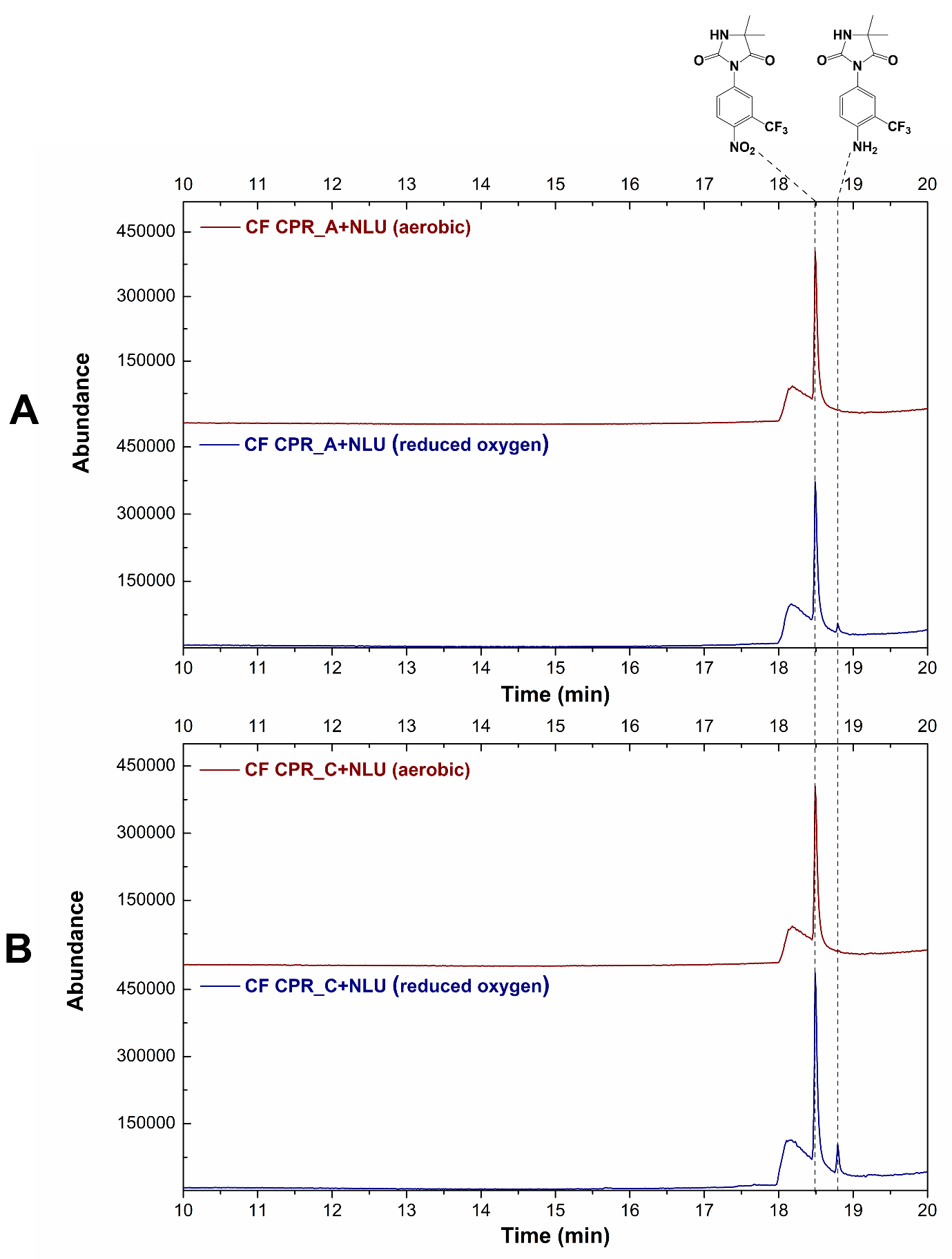


Table S1: Protein and gene sequences of *Cunninghamella elegans* NADPH: Cytochrome P450 Reductases

| **CPR** | **Protein sequence** | **Codon optimized gene sequence (for *P. pastoris*)** |
| --- | --- | --- |
| **CPR_A (g1631)** | >CPR_A_g1631_Cunninghamella_elegans  MLKSNNQVFEHQLMLVLGTISVGGLLWVAKGLLFNDSNKSSDQKPLIPDVKTMEKSEEEQEVGNFVKLMKEQNRKVIFFYGSQTGNAENYCYQLSKECKKRYGIQPMVADIETYDLKYLDLLTEDNLAVFIVSTYGEGDPTDSAINFWELIHNENPNFSKCDGNPQPLSKLRFFAFGLGNSTYEHFNAAVVGVDRELTRLGATRLGEIGKGDDDACLDDDFAHWQESFWPLFGEAVSQLIDGEADEGNHGDQYAYEVTDVNVDDNDGFYYQGELGSDRTQKLFDAKNPYPATVQIRDLTPATQDDRHCLHIDFDLADSGLTYKTGDHLGVWPINNELEVNLVSSIFGWNHDGILDKVISVKPTDPTGKVPFPQPTTLRTALRHYLDIAAIPSRSVFELLIPHVPEQIKAALQKIIDDKEVYNKLVVDEVRNFGQVLSHILTSNGYTDIENALAKVPINIIIECYTRLQPRYYSISSSSSESGSIVTATAVTLKYNPTPDRTVYGVNTNYLWSIYQKLNPSVANPNHPPYAIEGPHQAYFQKNGDTIITKLPVHIRQSTFRLPTDASTPVVMVGPGTGVAPFRGFVRERVYQKQVEKQQVGTTLLFFGCRRSNEDYLYADEWPELFNQLDNESRIINAFSRETDKKVYVQHRVRENGEEVWDLLANQGGYLYVCGDAKRMAKDITQTILDLAKHFGKLDDEAALAFVQGLRKAGRYQEDVWA | >CPR_A_g1631_Cunninghamella_elegans  ATGTTGAAGTCAAACAACCAAGTTTTCGAACATCAATTGATGTTGGTTTTGGGTACTATCTCAGTTGGTGGTTTGTTATGGGTTGCTAAGGGTTTGTTGTTTAATGATTCTAATAAGTCTTCAGATCAAAAGCCATTGATCCCAGATGTTAAGACTATGGAAAAATCTGAAGAAGAACAAGAAGTTGGTAACTTCGTTAAGTTGATGAAGGAACAAAATAGAAAAGTTATTTTCTTTTATGGTTCACAAACTGGTAACGCTGAAAACTACTGTTACCAATTGTCTAAGGAGTGTAAGAAAAGATACGGTATCCAACCAATGGTTGCAGATATCGAAACTTACGATTTGAAGTACTTGGATTTGTTGACAGAAGATAATTTGGCTGTTTTTATTGTTTCAACTTATGGTGAAGGTGACCCAACAGATTCTGCAATTAATTTCTGGGAATTGATCCATAACGAAAACCCAAACTTCTCAAAGTGTGATGGTAACCCACAACCATTGTCTAAGTTGAGATTTTTCGCTTTTGGTTTGGGTAACTCAACTTACGAACATTTCAATGCTGCAGTTGTTGGTGTTGATAGAGAATTAACTAGATTGGGTGCAACAAGATTAGGTGAAATTGGTAAAGGTGACGATGATGCTTGTTTGGATGATGATTTTGCACATTGGCAAGAATCATTTTGGCCATTATTTGGTGAAGCTGTTTCTCAATTGATTGATGGTGAAGCAGATGAAGGTAATCATGGTGACCAATATGCTTACGAAGTTACAGATGTTAACGTTGATGATAACGATGGTTTCTACTACCAGGGTGAATTGGGTTCTGATAGAACTCAAAAGTTGTTCGATGCTAAAAATCCATATCCAGCAACAGTTCAAATTAGAGATTTGACTCCAGCTACACAAGATGATAGACATTGTTTGCATATCGATTTCGATTTGGCAGATTCAGGTTTAACTTACAAAACAGGTGACCATTTGGGTGTTTGGCCAATTAATAACGAATTAGAAGTTAATTTGGTTTCTTCAATCTTCGGTTGGAACCATGATGGTATCTTGGATAAAGTTATTTCTGTTAAGCCAACTGATCCAACTGGTAAAGTTCCATTTCCACAACCAACTACATTGAGAACTGCTTTGAGACATTATTTGGATATTGCTGCAATCCCATCAAGATCTGTTTTCGAATTGTTGATCCCACATGTTCCAGAACAAATTAAAGCTGCATTGCAAAAGATTATCGATGATAAGGAAGTTTACAATAAGTTGGTTGTTGATGAAGTTAGAAACTTCGGTCAAGTTTTGTCACATATCTTGACTTCTAACGGTTACACAGATATCGAAAACGCTTTGGCAAAAGTTCCAATTAATATCATCATTGAATGTTACACAAGATTGCAACCAAGATACTACTCTATTTCTTCATCTTCATCTGAATCAGGTTCTATTGTTACTGCTACAGCAGTTACTTTGAAGTACAACCCAACTCCAGATAGAACAGTTTACGGTGTTAACACAAACTATTTGTGGTCAATCTATCAAAAGTTGAACCCATCTGTTGCTAACCCAAATCATCCACCATATGCTATTGAAGGTCCACATCAAGCATACTTCCAAAAGAATGGTGACACTATCATCACAAAGTTGCCAGTTCATATCAGACAATCAACTTTTAGATTGCCAACTGATGCTTCTACACCAGTTGTTATGGTTGGTCCAGGTACAGGTGTTGCACCTTTTAGAGGTTTCGTTAGAGAAAGAGTTTACCAAAAGCAAGTTGAAAAGCAACAAGTTGGTACTACATTGTTGTTTTTCGGTTGTAGAAGATCTAACGAAGATTATTTGTACGCTGATGAATGGCCAGAATTGTTTAATCAATTGGATAACGAATCAAGAATTATTAACGCATTTTCTAGAGAAACTGATAAGAAAGTTTACGTTCAACATAGAGTTAGAGAAAATGGTGAAGAAGTTTGGGATTTGTTAGCTAATCAAGGTGGTTATTTGTACGTTTGTGGTGACGCTAAAAGAATGGCAAAGGATATCACTCAAACAATCTTGGATTTGGCAAAACATTTTGGTAAATTGGATGATGAAGCTGCATTAGCTTTTGTTCAAGGTTTGAGAAAAGCAGGTAGATATCAAGAAGATGTTTGGGCT |
| **CPR_B (g4301)** | >CPR_B_g4301_Cunninghamella_elegans  MAQQSPAVIDTLDLILLGSIGLGTIAWFTRRQISERLFGTGQSNATSKPTTPQAPKRERNFVKVMEQQGRKVIFFYGSQTGTAEDFASRLAKQCSQKYGVSCMTADIEMYDLSYLDTLSEDSLVCFVMATYGEGEPTDNAVDFWEQFITDESPVFSQGGETLENLRYLMFGLGNKTYEHYNAVARILDKKLTGLGAKRIGERGEGDDDGSLEEDFLAWQESMWPTFCNALGVDENNAQQGPRQASYSVDELEEYQNDDVYFGELGTISKDSSRVVYDAKRPYNAPITTRELFNSSERHCLHVDIDISGTNLSYQTGDHVAMWPTNNEDEVLRLATILGLQEKLDTVISVKAIDPAAPKQNPFPVPTTYRAIFRHYIDICAPASRQSLMSFVEFAPTDAAKDLLKLLATDKDEYRLKVGEAVRNLGEVLELVSGKDAQPGSFSSVPFDLIVETIPRLQPRYYSISSSSKENPSIISATCVTLAYQPDPTPDRTVYGVNTNFLYRIHMQNNDDSVIQGLPKYDLAGPRKAFLNGQGQSHKLPIHIRRSQFKLPRNTSCPVIMIGPGTGVAPFRGFVRERALQKKEGKSVGPTILFFGNRHSQHDFLYSDEWPELFNTLGEDSKLITAFSRESEHKVYVQHRLEENGKEIWQLLEKGAYIYVCGDARNMARDVNQTFVNLAMEYGEKTEQKALDYVKSLRNTGRYQEDVWS | >CPR_B_g4301_Cunninghamella_elegans  ATGGCACAACAATCACCAGCTGTTATTGATACTTTGGATTTGATCTTGTTGGGTTCTATTGGTTTGGGTACTATTGCTTGGTTCACAAGAAGACAAATCTCAGAAAGATTGTTTGGTACTGGTCAATCAAATGCAACATCTAAACCAACTACACCACAAGCTCCAAAGAGAGAAAGAAACTTCGTTAAAGTTATGGAACAACAAGGTAGAAAAGTTATTTTCTTTTATGGTTCACAAACTGGTACAGCTGAAGATTTTGCATCTAGATTGGCTAAGCAATGTTCACAAAAGTACGGTGTTTCTTGTATGACTGCTGATATCGAAATGTACGATTTGTCTTACTTAGATACATTGTCTGAAGATTCATTGGTTTGTTTCGTTATGGCAACTTATGGTGAAGGTGAACCAACAGATAATGCTGTTGATTTCTGGGAACAATTCATTACTGATGAATCACCAGTTTTCTCTCAAGGTGGTGAAACATTAGAAAATTTGAGATACTTAATGTTCGGTTTGGGTAATAAGACTTACGAACATTACAACGCTGTTGCAAGAATCTTGGATAAGAAATTGACAGGTTTAGGTGCTAAAAGAATTGGTGAAAGAGGTGAAGGTGACGATGATGGTTCATTGGAAGAAGATTTCTTGGCATGGCAAGAATCTATGTGGCCAACTTTTTGTAATGCTTTGGGTGTTGATGAAAATAATGCACAACAAGGTCCAAGACAAGCTTCTTACTCAGTTGATGAATTGGAAGAATACCAAAACGATGATGTTTACTTCGGTGAATTGGGTACAATCTCTAAGGATTCTTCAAGAGTTGTTTACGATGCAAAGAGACCATACAATGCTCCAATCACTACAAGAGAATTGTTTAATTCTTCAGAAAGACATTGTTTGCATGTTGATATCGATATCTCAGGTACTAATTTGTCTTACCAAACAGGTGACCATGTTGCTATGTGGCCAACTAACAACGAAGATGAAGTTTTGAGATTGGCAACTATCTTGGGTTTGCAAGAAAAGTTGGATACAGTTATTTCTGTTAAAGCTATTGATCCAGCTGCACCAAAACAAAATCCATTTCCAGTTCCAACTACATACAGAGCAATTTTTAGACATTACATTGATATTTGTGCTCCAGCATCTAGACAATCATTGATGTCTTTCGTTGAATTTGCACCAACTGATGCTGCAAAGGATTTGTTGAAGTTGTTGGCTACAGATAAAGATGAATATAGATTGAAAGTTGGTGAAGCAGTTAGAAATTTGGGTGAAGTTTTGGAATTAGTTTCAGGTAAAGATGCTCAACCAGGTTCTTTTTCTTCAGTTCCATTCGATTTGATCGTTGAAACTATCCCAAGATTGCAACCAAGATACTACTCTATTTCTTCATCTTCAAAGGAAAACCCATCAATCATCTCTGCAACTTGTGTTACATTGGCTTATCAACCAGATCCAACTCCAGATAGAACAGTTTATGGTGTTAACACAAATTTCTTGTACAGAATCCATATGCAAAACAACGATGATTCAGTTATTCAAGGTTTGCCAAAATACGATTTGGCAGGTCCAAGAAAAGCATTTTTGAATGGTCAAGGTCAATCTCATAAGTTGCCAATCCATATCAGAAGATCACAATTCAAATTGCCAAGAAACACTTCTTGTCCAGTTATTATGATTGGTCCAGGTACAGGTGTTGCACCTTTTAGAGGTTTCGTTAGAGAAAGAGCTTTGCAAAAGAAAGAGGGTAAATCAGTTGGTCCAACTATCTTGTTTTTCGGTAACAGACATTCACAACATGATTTCTTGTATTCTGATGAATGGCCAGAATTGTTTAATACTTTGGGTGAAGATTCTAAGTTGATCACAGCTTTTTCAAGAGAATCTGAACATAAGGTTTACGTTCAACATAGATTAGAAGAAAACGGTAAAGAAATCTGGCAATTGTTAGAAAAGGGTGCTTACATCTATGTTTGTGGTGACGCAAGAAACATGGCTAGAGATGTTAACCAAACTTTCGTTAATTTGGCAATGGAATATGGTGAAAAGACTGAACAAAAGGCTTTGGATTACGTTAAGTCATTGAGAAATACAGGTAGATACCAAGAAGATGTTTGGTCT |
| **CPR_C (g7609)** | >CPR_C_ g7609_Cunninghamella_elegans  MTTRRSNNRVLGTYHLIVLGAIGLGTIAWFARNKFFGSSDTDDNKIISNSETSTTSSDTTAPKPNVPAKPERNFVKVMQQQGRRVIFFYGSQTGTAEDYASRLAKECSQKYGISAMTADIELYDLNYLDTLPEDNLVFFVMATYGEGEPTDNAVDFWELINDETPQFSQLANVEDKPLKNVRYLVFGLGNKTYEHYNSVGRTVDKKLTDFGATRIGERGEGDDDGSLEEDFLAWQETMWPAFCQALGIDENNIQLGPRQAAFKVEELTTFDDSSVYYGEIAENLKSKGKIVYDAKRPYNAPIQSRELFQDGGDRHCLHVDIDISGTNLNYQTGDHVAIWPTNNEIEVIRLASILGLKDKLDTVIMVNAVDSAASKQHPFPVPTTYRTIFRHYLDICSIASRQTLMSLVEYAPTEESKANLLRLSKDKDEYHVHVHDSVRNLGEVLQYVCGNNDHTDPEAHSGAFANVPFDLIIESISRLQPRYYSISSSSKEQPNIISATCVTLEYQPKPTPERTVYGVNTNYLWKIHSIVHQVDDGRSYPHYDIAGPRQQLFESSLSHAKIPIHVRRSQFKLPRNSKVPVIMVGPGTGVAPFRGFVRERALLKQKGQEVGPTVLFFGCRHSEKDFIYKDEWPELFNTLGEESQLITAFSRETDQKVYVQHRLKEYGQQMWDYIQQGAYIYVCGDAKNMAHDVNQAFIQFAQEFGGRDEVKANAFFKQLRNTGRYQEDVWS | >CPR_C_ g7609_Cunninghamella_elegans  ATGACTACAAGAAGATCTAACAACAGAGTTTTGGGTACTTACCATTTGATCGTTTTGGGTGCTATTGGTTTAGGTACAATCGCTTGGTTCGCAAGAAATAAGTTTTTCGGTTCTTCAGATACTGATGATAATAAGATCATCTCTAACTCAGAAACATCAACTACATCTTCAGATACTACAGCACCAAAACCAAATGTTCCAGCTAAGCCAGAAAGAAACTTCGTTAAAGTTATGCAACAACAAGGTAGAAGAGTTATTTTCTTTTATGGTTCTCAAACTGGTACAGCTGAAGATTACGCATCAAGATTGGCTAAGGAATGTTCTCAAAAGTACGGTATCTCAGCTATGACTGCAGATATCGAATTGTACGATTTGAACTACTTGGATACATTGCCAGAAGATAATTTGGTTTTCTTTGTTATGGCAACTTACGGTGAAGGTGAACCAACAGATAACGCTGTTGATTTCTGGGAATTGATTAATGATGAAACACCACAATTTTCTCAATTGGCTAACGTTGAAGATAAGCCATTGAAAAATGTTAGATATTTGGTTTTCGGTTTGGGTAATAAGACTTACGAACATTACAATTCTGTTGGTAGAACAGTTGATAAGAAATTGACTGATTTCGGTGCAACAAGAATCGGTGAAAGAGGTGAAGGTGACGATGATGGTTCATTGGAAGAAGATTTCTTGGCTTGGCAAGAAACTATGTGGCCAGCATTTTGTCAAGCTTTGGGTATCGATGAAAACAACATCCAATTAGGTCCAAGACAAGCTGCTTTTAAAGTTGAAGAATTGACTACATTCGATGATTCTTCAGTTTACTACGGTGAAATCGCTGAAAATTTGAAGTCTAAGGGTAAAATCGTTTACGATGCAAAGAGACCATACAATGCTCCAATTCAATCAAGAGAATTGTTTCAAGATGGTGGTGACAGACATTGTTTACATGTTGATATCGATATCTCTGGTACTAATTTGAACTATCAAACAGGTGACCATGTTGCAATTTGGCCAACTAACAACGAAATCGAAGTTATTAGATTGGCTTCAATCTTGGGTTTGAAGGATAAGTTGGATACAGTTATTATGGTTAACGCAGTTGATTCTGCTGCATCAAAACAACATCCATTTCCAGTTCCAACTACATACAGAACAATTTTTAGACATTACTTGGATATCTGTTCTATCGCTTCAAGACAAACTTTGATGTCTTTGGTTGAATACGCACCAACAGAAGAATCTAAAGCTAATTTGTTGAGATTGTCAAAGGATAAGGATGAATACCATGTTCATGTTCATGATTCTGTTAGAAATTTGGGTGAAGTTTTGCAATACGTTTGTGGTAACAATGATCATACAGATCCAGAAGCTCATTCAGGTGCTTTTGCAAATGTTCCATTCGATTTGATCATCGAATCTATTTCAAGATTACAACCAAGATACTACTCTATTTCTTCATCTTCAAAGGAACAACCAAACATCATCTCAGCTACTTGTGTTACATTGGAATATCAACCAAAACCAACTCCAGAAAGAACAGTTTATGGTGTTAACACTAACTACTTATGGAAGATCCATTCTATCGTCCATCAAGTTGATGATGGTAGATCATATCCACATTACGATATTGCAGGTCCAAGACAACAATTGTTCGAATCTTCATTGTCTCATGCTAAGATCCCAATCCATGTTAGAAGATCTCAATTCAAATTGCCAAGAAACTCAAAAGTTCCAGTTATTATGGTTGGTCCAGGTACTGGTGTTGCACCTTTTAGAGGTTTCGTTAGAGAAAGAGCTTTGTTGAAGCAAAAAGGTCAAGAAGTTGGTCCAACAGTTTTGTTTTTCGGTTGTAGACATTCTGAAAAGGATTTCATCTATAAGGATGAATGGCCAGAATTGTTTAATACTTTGGGTGAAGAATCTCAATTGATCACTGCATTTTCAAGAGAAACAGATCAAAAGGTTTACGTTCAACATAGATTGAAGGAATACGGTCAACAAATGTGGGATTACATTCAACAAGGTGCTTATATCTATGTTTGTGGTGACGCTAAAAATATGGCTCATGATGTTAACCAAGCTTTTATTCAATTTGCTCAAGAATTTGGTGGTAGAGATGAAGTTAAGGCTAACGCTTTCTTTAAGCAATTGAGAAATACTGGTAGATATCAAGAAGATGTTTGGTCT |
